# Retrospective BReast Intravoxel Incoherent Motion Multisite (BRIMM) multisoftware study

**DOI:** 10.3389/fonc.2025.1524634

**Published:** 2025-02-24

**Authors:** Dibash Basukala, Artem Mikheev, Xiaochun Li, Judith D. Goldberg, Nima Gilani, Linda Moy, Katja Pinker, Savannah C. Partridge, Debosmita Biswas, Masako Kataoka, Maya Honda, Mami Iima, Sunitha B. Thakur, Eric E. Sigmund

**Affiliations:** ^1^ Department of Radiology, Grossman School of Medicine, New York University, New York, NY, United States; ^2^ Department of Medical Physics, Memorial Sloan Kettering Cancer Center, New York, NY, United States; ^3^ Department of Population Health, Grossman School of Medicine, New York University, New York, NY, United States; ^4^ Department of Radiology, Vagelos College of Physicians and Surgeons, Columbia University Irving Medical Center, New York, NY, United States; ^5^ Department of Radiology, Memorial Sloan Kettering Cancer Center, New York, NY, United States; ^6^ Department of Bioengineering, University of Washington, Seattle, CA, United States; ^7^ Department of Radiology, School of Medicine, University of Washington, Seattle, WA, United States; ^8^ Department of Diagnostic Imaging and Nuclear Medicine, Kyoto University Graduate School of Medicine, Kyoto, Japan; ^9^ Department of Diagnostic Radiology, Kansai Electric Power Hospital, Osaka, Japan; ^10^ Department of Fundamental Development for Advanced Low Invasive Diagnostic Imaging, Graduate School of Medicine, Nagoya University, Nagoya, Japan; ^11^ Center for Advanced Imaging Innovation and Research, New York University, New York, NY, United States

**Keywords:** IVIM, DWI, breast cancer, diagnosis, multisite, multisoftware, radiomics, robust

## Abstract

**Introduction:**

The intravoxel incoherent motion (IVIM) model of diffusion weighted imaging (DWI) provides imaging biomarkers for breast tumor characterization. It has been extensively applied for both diagnostic and prognostic goals in breast cancer, with increasing evidence supporting its clinical relevance. However, variable performance exists in literature owing to the heterogeneity in datasets and quantification methods.

**Methods:**

This work used retrospective anonymized breast MRI data (302 patients) from three sites employing three different software utilizing least-squares segmented algorithms and Bayesian fit to estimate 1_st_ order radiomics of IVIM parameters perfusion fraction (*f_p_*), pseudo-diffusion (*D_p_*) and tissue diffusivity (*D_t_*). Pearson correlation (*r*) coefficients between software pairs were computed while logistic regression model was implemented to test malignancy detection and assess robustness of the IVIM metrics.

**Results:**

*D_t_* and *f_p_* maps generated from different software showed consistency across platforms while *D_p_* maps were variable. The average correlation between the three software pairs at three different sites for 1_st_ order radiomics of IVIM parameters were *D_t_min*/*D_t_max*/*D_t_mean*/*D_t_variance*/*D_t_skew*/*D_t_kurt*: 0.791/0.891/0.98/0.815/0.697/0.584; *f_p_max*/*f_p_mean*/*f_p_variance*/*f_p_skew*/*f_p_kurt*: 0.615/0.871/0.679/0.541/0.433; *D_p_max*/*D_p_mean*/*D_p_variance*/*D_p_skew*/*D_p_kurt*: 0.616/0.56/0.587/0.454/0.51. Correlation between least-squares algorithms were the highest. *D_t_mean* showed highest area under the ROC curve (AUC) with 0.85 and lowest coefficient of variation (CV) with 0.18% for benign and malignant differentiation using logistic regression. *D_t_* metrics were highly diagnostic as well as consistent along with *f_p_* metrics.

**Discussion:**

Multiple 1_st_ order radiomic features of *D_t_* and *f_p_* obtained from a heterogeneous multi-site breast lesion dataset showed strong software robustness and/or diagnostic utility, supporting their potential consideration in controlled prospective clinical trials.

## 1 Introduction

Breast cancer remains a leading cause of cancer-related deaths in women in the U.S (1). Diffusion weighted MRI (DW-MRI or DWI) provides biomarkers for cancer diagnosis and characterization (2–6), and has been demonstrated to distinguish benign and malignant breast lesions (7–10) without using contrast agents.

Intravoxel incoherent motion (IVIM) (11–13), an advanced DWI technique allows simultaneous quantification of diffusion and perfusion properties of the tissue. IVIM is sensitive to cellularity and microvascular flow and there is a growing evidence base of its clinical utility for both diagnostic and prognostic goals in the setting of breast cancer (14–18). IVIM uses a biexponential function (see Equation 1.1) to describe the diffusion signal decay over different b-values to estimate tissue diffusivity (*D_t_*), pseudo-diffusion (*D_p_*), and perfusion fraction (*f_p_*). These IVIM coefficients serve as biomarkers for the identification of different tumor biologic characteristics. Specifically, *D_t_* is sensitive to restrictions to Brownian water motion such as cell membranes, fibrosis, or macromolecules. *f_p_* reflects the volume fraction of faster microcirculation, often originating from the microvascular space. Finally, *D_p_* reflects the apparent diffusion process in the microcirculatory space which is impacted by both fluid flow speed and vascular architecture. For the specific case of breast cancer, malignant tumors often exhibit lower *D_t_* values due to higher cellularity, higher *f_p_* due to higher vascularity and lower *D_p_* due to slower blood velocity compared to benign lesions.

One obstacle to clinical implementation is the variability of algorithms and tools used to determine the IVIM metrics, which can introduce corresponding variability in clinical performance. For example, most IVIM parameters’ estimation is based on nonlinear least squares (19, 20), segmented least squares fitting (17, 21–23), or the Bayesian (24–27) approach. More recently, deep learning (DL) based approaches have gained significant attention for their mitigation of acquisition (28) and noise-induced variability compared to traditional methods, especially for *f_p_* and *D_p_* (29–32). Furthermore, most prior studies calculated IVIM coefficients based on the mean values within the region of interest (ROI), whereas radiomic features of IVIM maps may potentially provide more information and capture tumor heterogeneity (33–35).

Nevertheless, differences in patient cohorts, scanners, acquisition protocols, and analysis algorithms (36–39) contribute to variable diagnostic performance between studies and can dilute the potential of the IVIM biomarkers for more widespread adoption in clinical trials or daily practice (8, 10). A retrospective cross-sectional view of a large subset of available clinical data from patients presenting with suspicious lesions, acquired at different sites analyzed with widely used software platforms, may be illuminating to highlight the software dependency of IVIM parameters as well as the most robust and diagnostic 1^st^ order radiomic features in the IVIM dataset and guide future harmonization efforts in multi-center trials.

## 2 Materials and methods

This study evaluated retrospective anonymized breast MR imaging data from three different sites. The patients were scanned using 1.5 T or 3 T scanners at each site (Site A: GE Healthcare, Waukesha, Wisconsin, USA, 1.5 T and 3.0 T; Site B: Philips Healthcare, Best, the Netherlands, 3.0 T; and Site C: Siemens trio, Siemens Healthcare, Erlangen, Germany, 3.0 T). Details of each acquisition protocol and studied cohort are listed in **Tables 1**, **2**. Criteria for evaluation varied between sites. Site A included the patients who underwent breast MRI screening when they were suspicious of Breast Imaging Reporting and Data System (BI-RADS) 4, 5 and/or cancer-proven BI-RADS 6 lesions. Site B included patients underwent breast MRI screening and had BI-RADS 4, 5 lesions detected. Site C included the patients who underwent breast MRI when they were known to have or were suspected of having breast carcinoma. This included patients with BI-RADS categories 2-5 lesions.

**Table 1:** MRI system and acquisition parameters used at each site in the multicenter study. Resolutions are given in acquired and reconstructed voxel sizes.

		Site A		Site B	Site C
MRI system	Vendor	GE 1.5 T/3 T		Philips 3 T	Siemens Trio 3 T
**Resolution (mm)**		2.2 - 2.8/2.2 - 2.8/4 Recon. 1.1 - 1.4/1.1 - 1.4/4		1.8/1.8/4 Recon. 1.3/1.3/4	2.0/2.0/3
**Echo time (ms)**		96.2		59.2	88.4
**Repetition time (ms)**		6000		4987.5	4700
**b-values (s/mm^2^)**		9	0,30,60,90,120,250, 400,600,800	0,100,600,800,1000	0,5,10,20,30,50,70,100,200,400,600,800,1000
		24	0,30,60,90,120,400, 600,800,1000		
		10	0,30,60,90,120,250, 450,600,800,1000		
		14	0,30,60,90,120,250, 400,600,800,1000		
		1	0,10,30,60,90,120, 200,400,600,800, 1000		

**Table 2:** Number of patients with breast lesions from multiple centers along with average age at each site.

		Site A			Site B			Site C		
		N	ROI Size		N	ROI Size		N	ROI Size	
			Voxels	cm^3^		Voxels	cm^3^		Voxels	cm^3^
**Lesions**	**Benign**	12	201 ± 143	0.97 ± 0.69	70	51 ± 101	0.32 ± 0.63	38	33 ± 40	0.39 ± 0.47
	**Malignant**	46	1363 ± 1644	6.6 ± 7.96	19	34 ± 28	0.21 ± 0.18	117	56 ± 69	0.67 ± 0.83
	**Total**	58	1123 ± 1537	5.43 ± 7.44	89	47 ± 91	0.29 ± 0.57	155	50 ± 64	0.6 ± 0.77
**Age (yrs)**		48.26 ± 9.61			46.12 ± 11.34			57.03 ± 15.25		

Voxel count, size values (in cubic centimeters) and age are given in mean ± standard deviation.

Lesion conspicuity was assessed by radiologists on either b0 or b>0 DWI images, in comparison with dynamic contrast enhanced (DCE) MRI at Site A, Site B and Site C. Referencing the accompanying DCE MRI, ROIs were drawn on either b0 or b>0 DWI images in consultation with the respective team radiologist. At Site A ROIs were prescribed on all lesion slices, while for Sites B and C only the central slice of largest cross section was prescribed. The ROI contains at least 3 voxels, and no obvious artifacts were included at all sites as per the guidelines from the European Society of Breast Radiology (EUSOBI) (8). Single lesion per patient was used at all sites. In addition, lesions were histologically confirmed (Site A and Site B), or sometimes based on radiologist reports, and based on stability on imaging for more than 18 months for benign lesions at Site C.

### 2.1 Data analysis

IVIM data from all sites were independently analyzed using three software packages: a shareware tool with least-squares segmented fitting (Firevoxel, https://firevoxel.org/ (Software a)), an MR vendor research software package with least-squares segmented fitting (Siemens MR Body Diffusion Toolbox from Siemens Healthineers (Software b)) and a commercial software package with Bayesian fit algorithm Olea Sphere (Software c).

IVIM parameters were estimated from a fit of all acquired b-values (see **Table 1**) to a biexponential decay:


1.1 $$\frac{S}{S_{0}}=f_{p}\exp\left(-b\cdot D_{p}\right)+\left(1-f_{p}\right)\exp\left(-b\cdot D_{t}\right)$$

IVIM parameters *f_p_*, *D_p_* and *D_t_* were calculated from the voxels in the lesion ROI using each software tool. Histogram analysis of parametric maps generated by each software was also performed within a separate module for histogram generation in Firevoxel (100 bins, *f_p_*: 0 – 1, *D_p_*: 0 – 0.1 mm^2^/s and *D_t_*: 0 – 0.003 mm^2^/s) to estimate 1^st^ order radiomic features from each parameter: mean/minimum/maximum/variance/skewness/kurtosis. This single histogram module was used to limit the software differences to that in IVIM estimation alone.

### 2.2 Statistical analysis

The Pearson correlation (*r*) coefficient of IVIM parameters for the 1^st^ order radiomic features was computed between each software pair at each site separately. The average correlation coefficient and coefficient of variation (CV) over all software pairs and sites was computed for each metric and ranked in numerical order to assess the consistency of performance of a clinical task. The intraclass correlation coefficient (ICC) was also computed for the agreement among three software for the IVIM metrics at each site. Additionally, Bland-Altman analysis (40) of IVIM parameters for the 1^st^ order radiomic features was also carried out between each software pair at each site separately. Measures of absolute difference mean, absolute difference standard deviation, and CV (%) were derived from each software pair comparison.

Within the context of each software, each IVIM metric was tested for benign/malignant differentiation, separately for each software, using logistic regression for all three sites’ data together, with each variable adjusted by site (coefficient and intercept). In addition, we also performed leave-one-patient-out (LOU) cross validation for each IVIM metric for the logistic regressions adjusted by site for each software. The area under the ROC curve (AUC) and standard error (SE) were quantified for each software separately. An average of AUCs (separately for original and LOU analysis) across software was computed for each IVIM metric. CVs of the three AUCs from each software were computed for benign and malignant differentiation. These average metrics were then ranked in numerical order for assessment of consistency of performance of a clinical task. Additionally, AUCs from all pairs of software were separately compared with DeLong’s test. Statistical analysis was performed using MATLAB software for Bland-Altman analysis and R 4.2 software for ICC and logistic regression.

## 3 Results

The study included 58, 89 and 155 patients from Site A, Site B, and Site C respectively. Site A, Site B and Site C included 79.3%, 21.4%, and 75.5% of patients with malignant lesions respectively, with each patient contributing one lesion. **Table 2** shows the distribution of the patients including the number of biopsy-confirmed benign/malignant lesions across sites in this retrospective multicenter study along with ROI size. The number of voxels per ROI ranged from 47 ± 91 (Site B) to 50 ± 64 (Site C), and up to 1123 ± 1537 (Site A). In addition, average age across sites is also reported.

**Figure 1:**
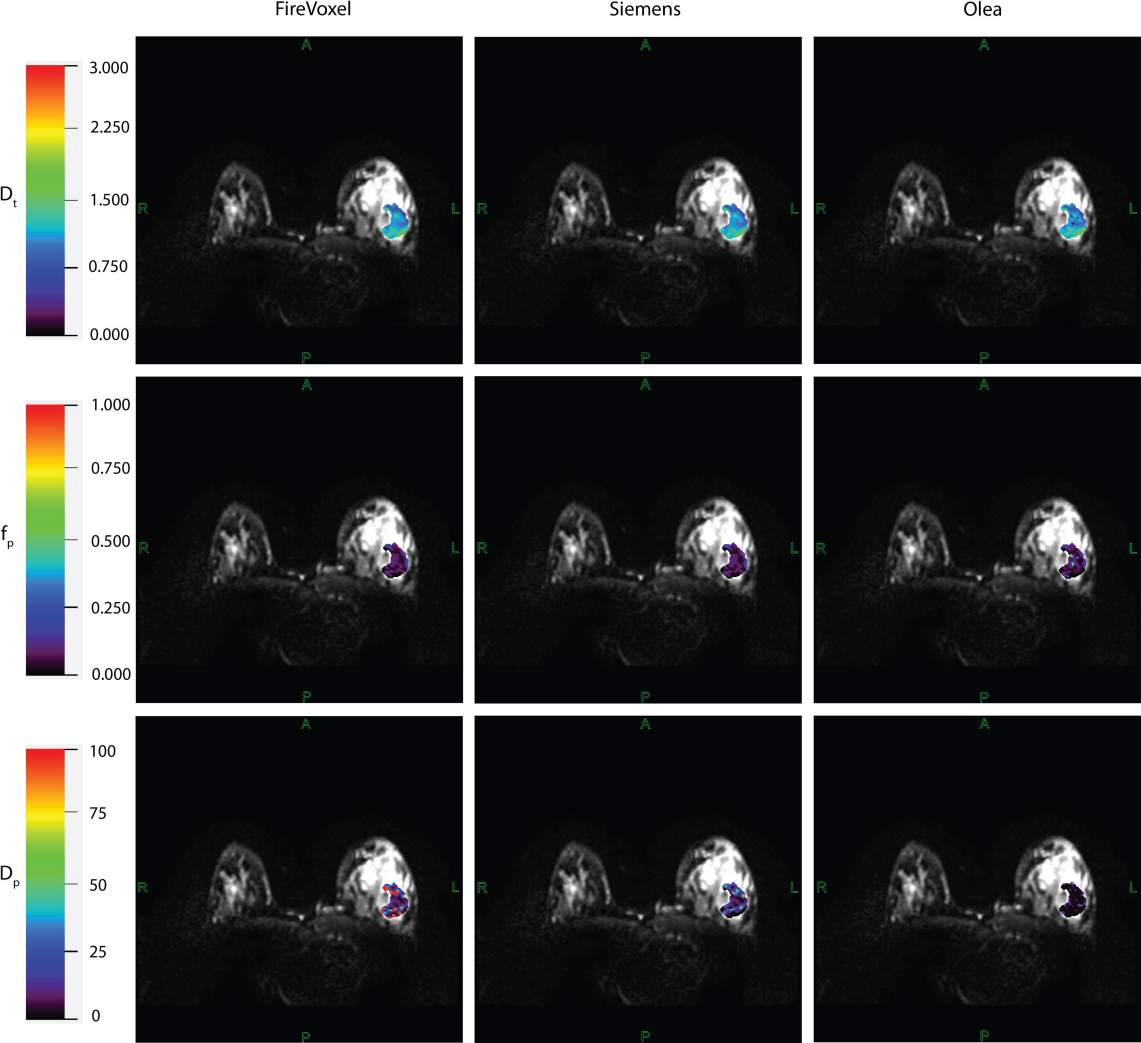
IVIM parametric maps overlaid on raw DWI images in a patient with malignant breast lesion for Site A. IVIM parameters tissue diffusivity (*D_t_*), perfusion fraction (*f_p_*) and pseudodiffusivity (*D_p_*) obtained from Firevoxel, Siemens and Olea software in the breast lesion. *D_t_* maps and *f_p_* maps are the most consistent across software platforms, while *D_p_* maps show the most variability with fit algorithms. *D_t_* and *D_p_* are given in units of 10 ^-3^ mm^2^/s.

**Figure 2:**
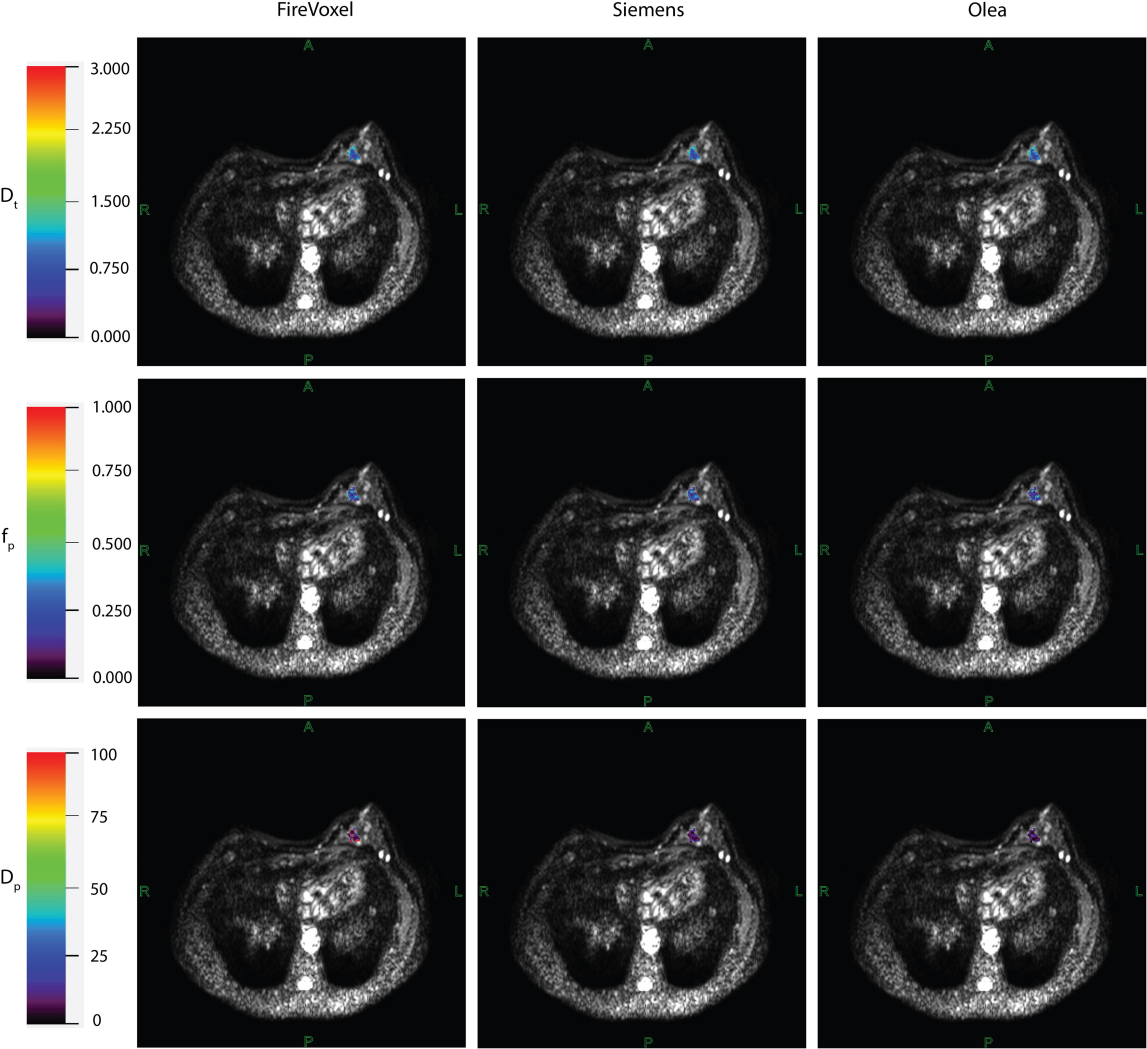
IVIM parametric maps overlaid on raw DWI images in a patient with malignant breast lesion for Site B. IVIM parameters tissue diffusivity (*D_t_*), perfusion fraction (*f_p_*) and pseudodiffusivity (*D_p_*) obtained from Firevoxel, Siemens and Olea software in the breast lesion. *D_t_* maps and *f_p_* maps are the most consistent across software platforms, while *D_p_* maps show the most variability with fit algorithms. *D_t_* and *D_p_* are given in units of 10 ^-3^ mm^2^/s.

**Figure 3:**
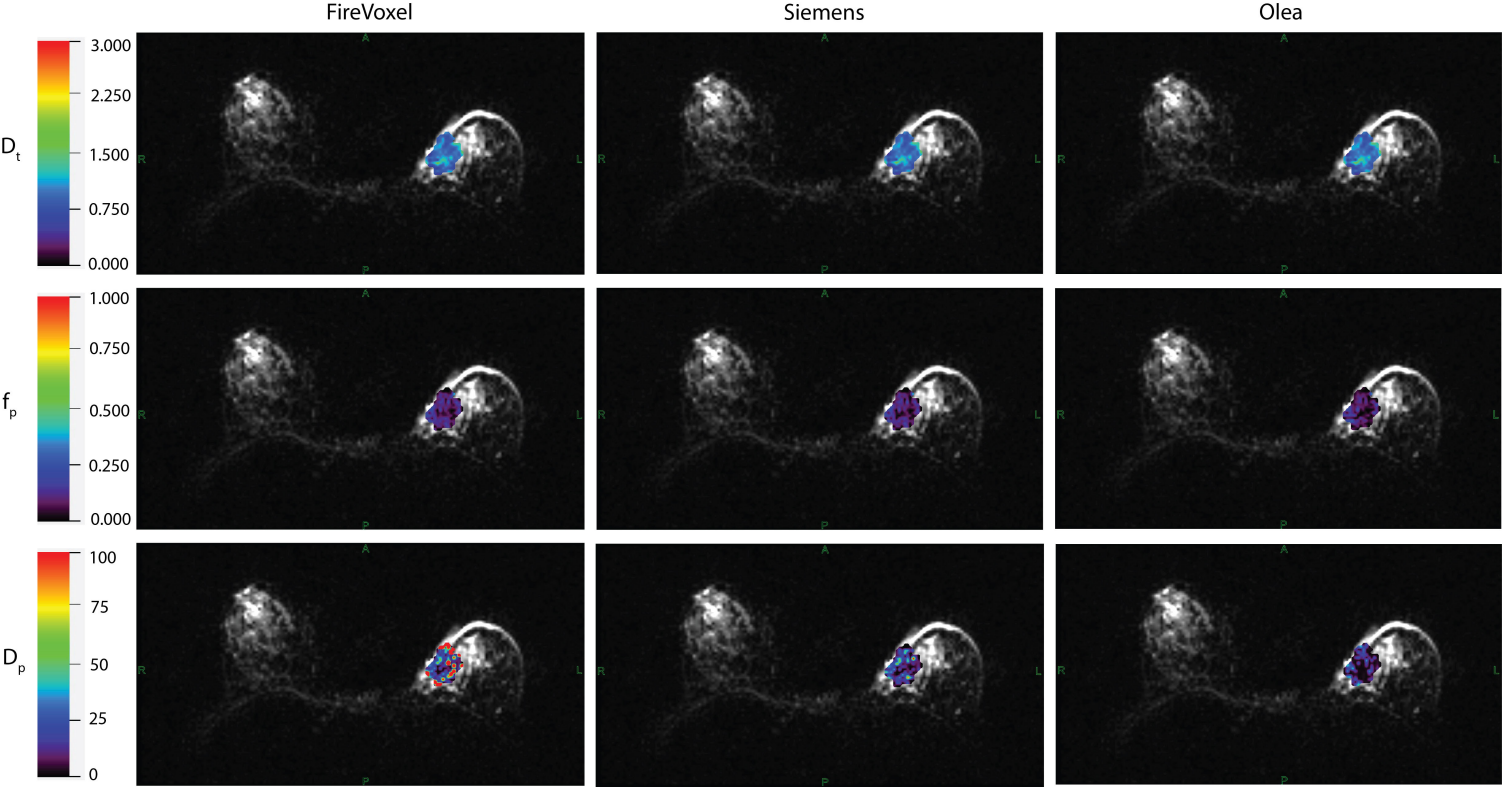
IVIM parametric maps overlaid on raw DWI images in a patient with malignant breast lesion for Site C. IVIM parameters tissue diffusivity (*D_t_*), perfusion fraction (*f_p_*) and pseudodiffusivity (*D_p_*) obtained from Firevoxel, Siemens and Olea software in the breast lesion. *D_t_* maps and *f_p_* maps are the most consistent across software platforms, while *D_p_* maps show the most variability with fit algorithms. *D_t_* and *D_p_* are given in units of 10 ^-3^ mm^2^/s.

Example IVIM parameter maps obtained from each software for malignant lesions from Site A, Site B and Site C are shown in **Figure 1**, **Figures 2**, **3** respectively. Example benign breast lesions are shown in **Supplementary Figure S1-S3**. Overall *D_t_* maps and *f_p_* maps show consistency while *D_p_* maps exhibit the most variability across the software platforms. The average fractions of utilized voxels per lesion (i.e. having values within the prescribed histogram ranges) were as follows. *D_t_* utilized 99.72%/99.88%/99.87% of lesion voxels at Site A; 100%/100%/100% at Site B; 99.99%/99.95%/100% at Site C using Software a/b/c. *D_p_* utilized 77.51%/99.77%/100% of lesion voxels at Site A; 80.58%/100%/100% at Site B; 59.78%/99.23%/100% at Site C using Software a/b/c while *f_p_* utilized 100% of lesion voxels at all sites using Software a, b, c. The mean IVIM parameter values for benign and malignant lesions at Site A, Site B and Site C are shown in **Table 3** which clearly indicates the consistency of *D_t_* and *f_p_* values across all software platforms except for *f_p_* at Site B. Mean *f_p_* values were found to be somewhat variable between least squares segmented fitting and Bayesian fitting at Site B.

**Table 3:** Mean IVIM parameter values for benign and malignant lesion employing Software (a, b, c) at Site A, Site B and Site C.

		Site A			Site B			Site C		
		a	b	c	a	b	c	a	b	c
***D_t_***	Benign	1.26 ± 0.29	1.26 ± 0.27	1.25 ± 0.24	1.04 ± 0.33	1.05 ± 0.31	1.12 ± 0.31	1.36 ± 0.4	1.31 ± 0.4	1.31 ± 0.35
	Malignant	1.2 ± 0.39	1.19 ± 0.38	1.18 ± 0.38	0.88 ± 0.29	0.88 ± 0.28	0.96 ± 0.31	0.93 ± 0.29	0.9 ± 0.28	0.9 ± 0.29
***f_p_***	Benign	15.79 ± 9.87	14.42 ± 8.87	16.34 ± 8.36	18.62 ± 7.49	16.47 ± 7.41	11.06 ± 7.46	12.77 ± 6.95	11.78 ± 7	11.08 ± 6.77
	Malignant	13.85 ± 5.02	13.03 ± 4.51	14.34 ± 4.85	20.66 ± 7.15	19.32 ± 7.85	14.03 ± 6.79	11.41 ± 4.91	10.21 ± 3.9	9.4 ± 4.61
***D_p_***	Benign	10.57 ± 3.83	9 ± 3.54	5.46 ± 2.63	6.07 ± 2.34	5.25 ± 2.99	6.03 ± 3.51	18.02 ± 8.47	10.87 ± 4.81	6.77 ± 4.12
	Malignant	12.02 ± 3.12	9.85 ± 3.17	7.85 ± 2.91	7.06 ± 2.02	7.27 ± 3.24	8.62 ± 4.05	16.23 ± 5.94	9.84 ± 4.03	11.43 ± 4.64

a: Firevoxel; b: Siemens; c: Olea.

Perfusion fraction (*f_p_*) is given in %, while pseudo-diffusion (*D_p_*) and tissue diffusivity (*D_t_*) are given in units of 10 ^-3^ mm^2^/s.

Data are given in mean ± standard deviation.

**Figure 4:**
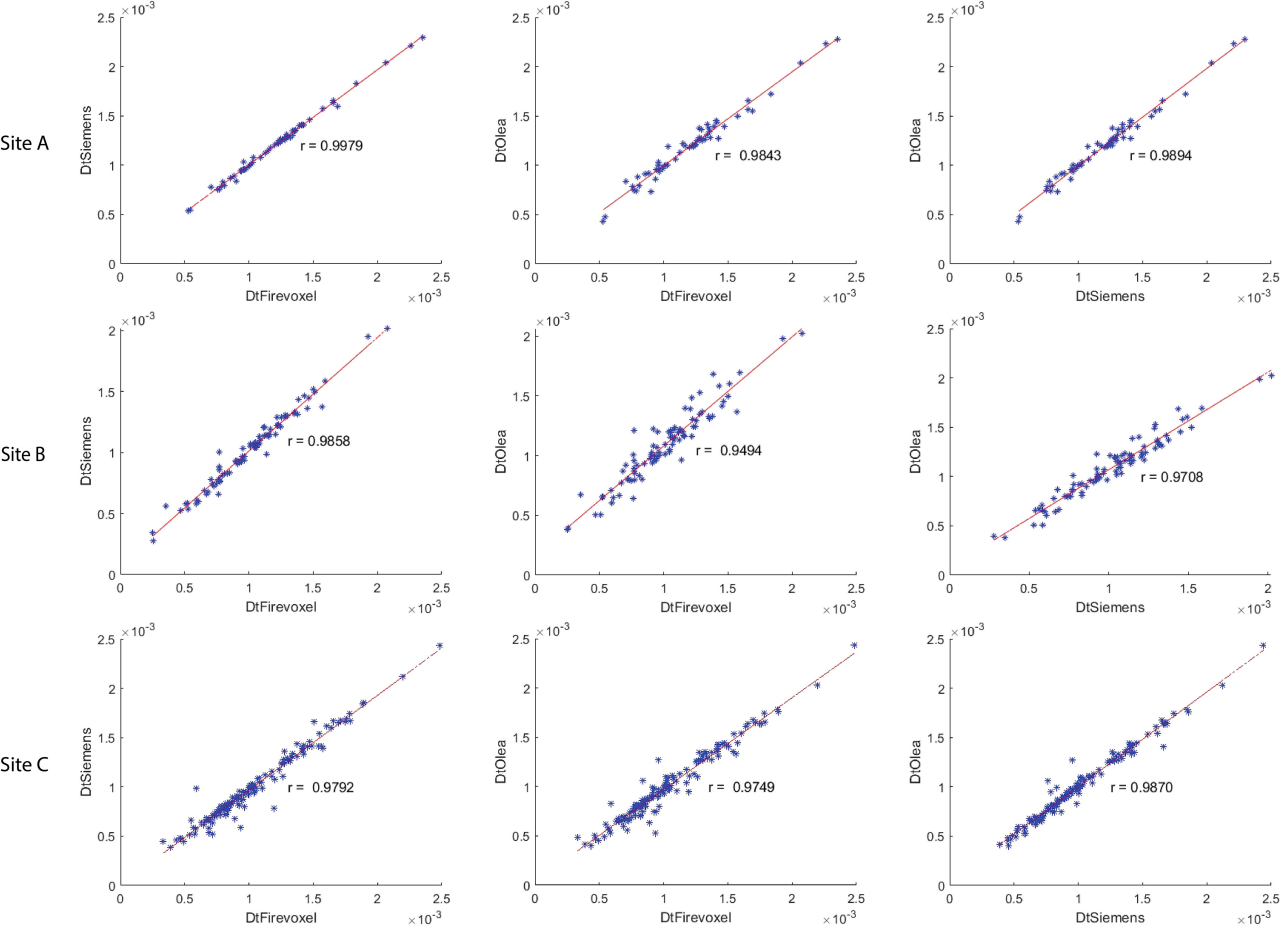
Correlation coefficient between Firevoxel, Siemens and Olea for mean of tissue diffusivity (*D_t_*) at Site A, Site B and Site C. Comparisons shown left to right: Firevoxel *vs*. Siemens, Firevoxel *vs*. Olea, and Siemens *vs*. Olea. Least-squares segmented algorithms (Firevoxel, Siemens) and Bayesian algorithms (Olea) show excellent agreement. *D_t_* is given in unit of mm^2^/s.

**Figure 5:**
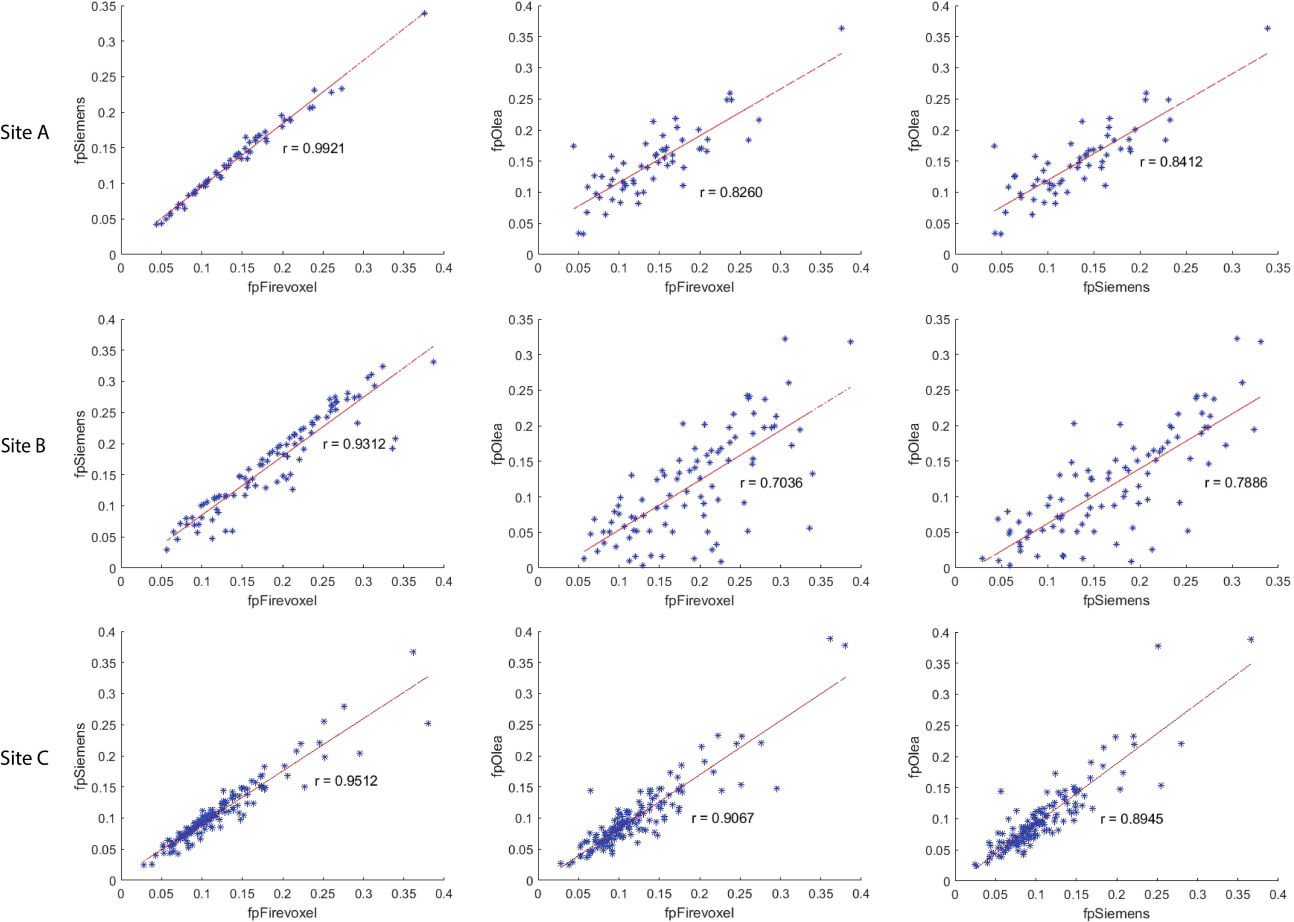
Correlation coefficient between Firevoxel, Siemens and Olea for mean of perfusion fraction (*f_p_*) at Site A, Site B and Site C. Comparisons shown left to right: Firevoxel *vs*. Siemens, Firevoxel *vs*. Olea, and Siemens *vs*. Olea. Least-squares segmented algorithms (Firevoxel, Siemens) show the highest agreement while correlation between least-squares and Bayesian algorithms (Olea) is somewhat less.

**Figure 6:**
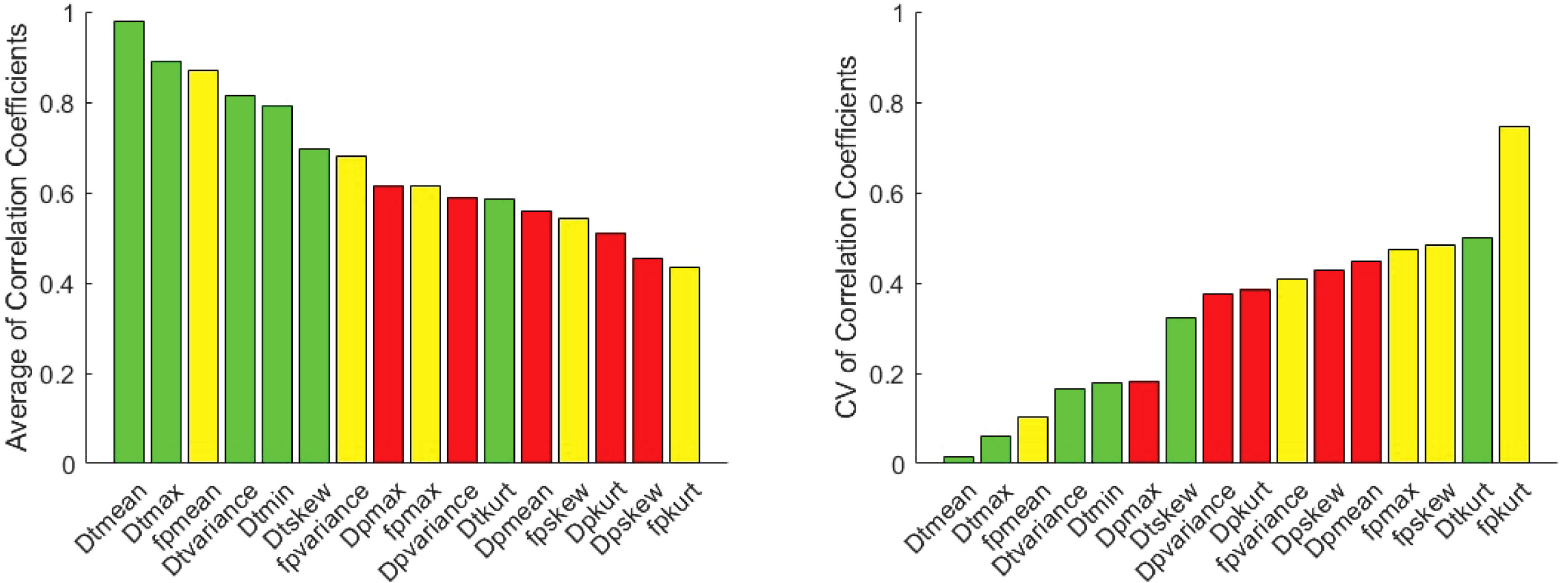
Average Pearson correlation coefficients of 1^st^ order radiomic features of *f_p_* (yellow), *D_t_* (green) and *D_p_* (red) between software pairs at Site A, Site B and Site C along with coefficient of variation (CV) of correlation coefficients. Highest correlations are observed for mean *D_t_* (lowest CV) and *f_p_* metrics as well as other *D_t_* radiomics.

The correlation coefficient of IVIM parameters between each software pair for 1^st^ order radiomic features at each site is shown in **Supplementary Table S1** along with ICC values. Correlations between least-squares segmented fitting algorithms are generally higher than those between least squares and Bayesian algorithms. The average correlation between the three software at three different sites for 1^st^ order radiomic features mean/maximum/variance/skewness/kurtosis were *f_p_* (*r* = 0.871/0.615/0.679/0.541/0.433), *D_p_* (*r* = 0.56/0.616/0.587/0.454/0.51) and *D_t_* (*r* = 0.98/0.891/0.815/0.697/0.584) respectively while that for *D_t_min* was 0.791. The correlations between the three software for mean *D_t_* at Site A, Site B and Site C are shown in **Figure 4**; excellent correlation observed between least-squares segmented algorithms (Firevoxel and Siemens) and Bayesian algorithms (Olea) at each site. Similarly, the correlations between the three software for mean *f_p_* at Site A, Site B and Site C are shown in **Figure 5**; strongest correlation observed between least-squares segmented algorithms (Firevoxel and Siemens) at each site. **Figure 6** shows the average of correlation coefficients of 1^st^ order radiomic features of *f_p_*, *D_t_* and *D_p_* across all software and sites along with CV of correlation coefficients. In general, *D_t_* radiomics showed the highest average software correlation along with mean *f_p_* while *D_t_mean* showed the lowest CV. Additionally, Bland-Altman analysis of IVIM parameters between each Software (a, b, c) pair for 1^st^ order radiomics at each site is shown in **Supplementary Table S2**. Bland-Altman plots between the three software for mean *D_t_* and mean *f_p_* at Site A, Site B and Site C are shown in **Supplementary Figures S4**, **S5**.

**Figure 7:**
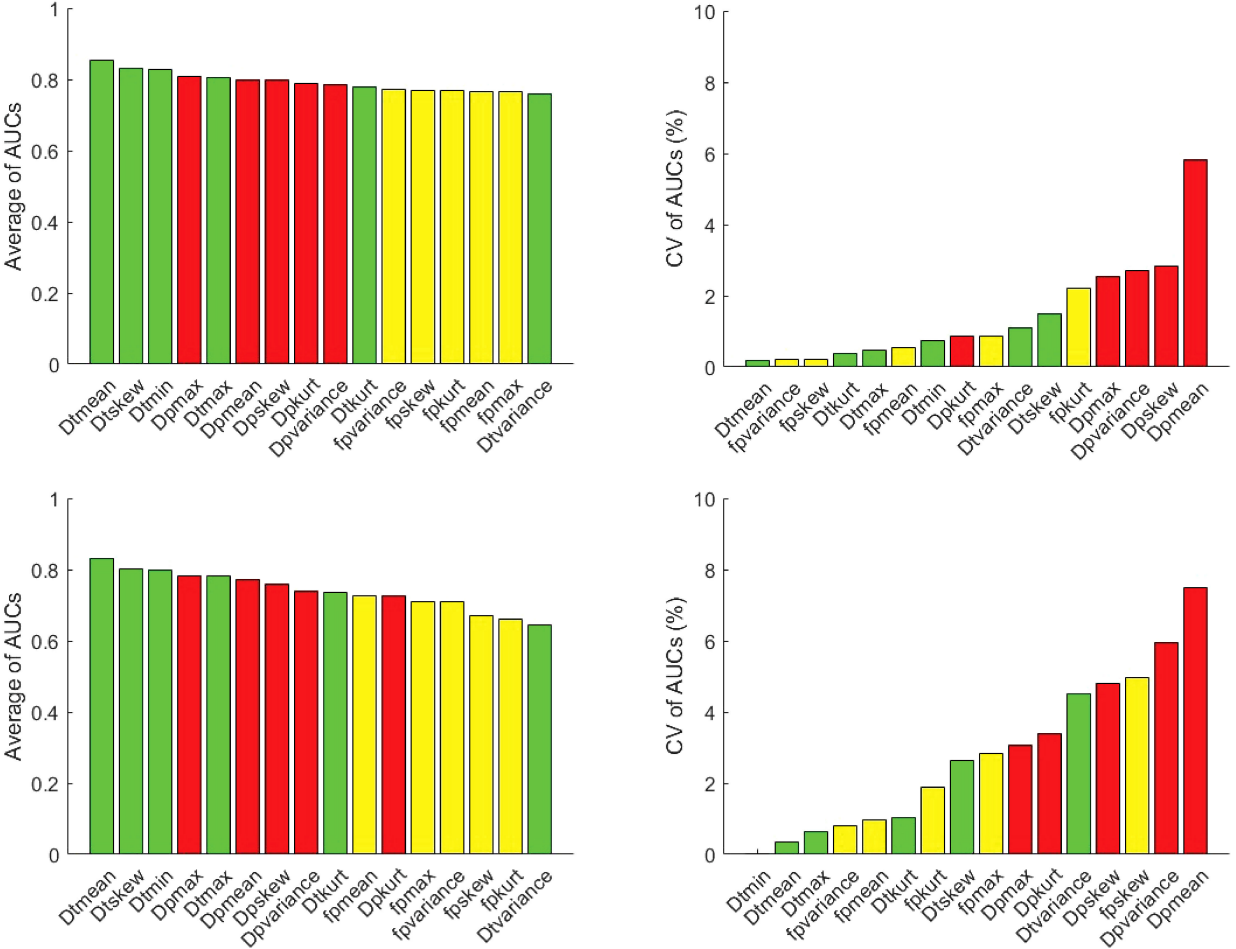
Average area under the ROC curve (AUC) and coefficient of variation (CV) of AUC for benign and malignant differentiation via metrics of *f_p_* (yellow), *D_t_* (green) and *D_p_* (red) using logistic regression (top row) and leave-one-patient-out (LOU) cross validation (bottom row). *D_t_* metrics generally show the highest average and most consistent performance for the benign/malignant task, and several *f_p_* metrics (e.g. mean and variance) show high consistency among software.

No pair of parameter AUCs from different software were significantly different (*p*>0.05). Regarding the pooled site analyses, the AUC with SE for benign and malignant differentiation for different IVIM metrics employing different software from three sites using logistic regression and LOU cross validation is shown in **Table 4** while the average of AUC as well as CV of AUC (%) for benign and malignant differentiation is shown in **Figure 7**. For both AUC analyses, mean, minimum, maximum and skewness of *D_t_* showed the highest average AUC followed by *D_p_* metrics for the benign/malignant task while mean and variance of *f_p_* along with several *D_t_* radiomics showed high consistency among software. LOU AUCs showed a similar ranking of performance to logistic regression AUC with a few exceptions (such as higher ranking of *f_p_* mean), with slightly lower and more spread values of average AUC, and higher and more spread values of CV of AUC.

**Table 4:** Area under the ROC curve (AUC) with standard error (SE) using logistic regression and leave-one-patient-out (LOU) cross validation AUC with SE for benign and malignant differentiation for different IVIM metrics using Software (a, b, c) from Site A, Site B and Site C.

	Software	AUC (SE)	LOU AUC (SE)		Software	AUC (SE)	LOU AUC (SE)		Software	AUC (SE)	LOU AUC (SE)
***D_t_min***	a	0.82 (0.02)	0.8 (0.03)								
	b	0.82 (0.02)	0.8 (0.03)								
	c	0.83 (0.02)	0.8 (0.03)								
***D_t_max***	a	0.8 (0.03)	0.78 (0.03)	***f_p_max***	a	0.76 (0.03)	0.7 (0.03)	***D_p_max***	a	0.81 (0.02)	0.78 (0.03)
	b	0.81 (0.02)	0.79 (0.03)		b	0.77 (0.03)	0.73 (0.03)		b	0.79 (0.03)	0.76 (0.03)
	c	0.8 (0.03)	0.78 (0.03)		c	0.76 (0.03)	0.69 (0.03)		c	0.83 (0.02)	0.81 (0.02)
***D_t_mean***	a	0.85 (0.02)	0.83 (0.02)	***f_p_mean***	a	0.77 (0.03)	0.73 (0.03)	***D_p_mean***	a	0.77 (0.03)	0.74 (0.03)
	b	0.85 (0.02)	0.83 (0.02)		b	0.76 (0.03)	0.72 (0.03)		b	0.78 (0.03)	0.73 (0.03)
	c	0.86 (0.02)	0.84 (0.02)		c	0.77 (0.03)	0.73 (0.03)		c	0.85 (0.02)	0.84 (0.02)
***D_t_variance***	a	0.75 (0.03)	0.62 (0.03)	***f_p_variance***	a	0.77 (0.03)	0.72 (0.03)	***D_p_variance***	a	0.78 (0.03)	0.72 (0.03)
	b	0.77 (0.03)	0.68 (0.03)		b	0.77 (0.03)	0.71 (0.03)		b	0.77 (0.03)	0.71 (0.03)
	c	0.76 (0.03)	0.64 (0.03)		c	0.77 (0.03)	0.7 (0.03)		c	0.81 (0.02)	0.79 (0.03)
***D_t_skew***	a	0.82 (0.02)	0.78 (0.03)	***f_p_skew***	a	0.77 (0.03)	0.66 (0.03)	***D_p_skew***	a	0.8 (0.03)	0.76 (0.03)
	b	0.82 (0.02)	0.8 (0.03)		b	0.77 (0.03)	0.64 (0.03)		b	0.78 (0.03)	0.72 (0.03)
	c	0.85 (0.02)	0.83 (0.02)		c	0.77 (0.03)	0.71 (0.03)		c	0.82 (0.02)	0.79 (0.03)
***D_t_kurt***	a	0.78 (0.03)	0.74 (0.03)	***f_p_kurt***	a	0.79 (0.03)	0.65 (0.03)	***D_p_kurt***	a	0.8 (0.03)	0.75 (0.03)
	b	0.78 (0.03)	0.73 (0.03)		b	0.75 (0.03)	0.68 (0.03)		b	0.78 (0.03)	0.73 (0.03)
	c	0.78 (0.03)	0.74 (0.03)		c	0.77 (0.03)	0.66 (0.03)		c	0.79 (0.03)	0.7 (0.03)

a: Firevoxel; b: Siemens; c: Olea.

## 4 Discussion

Our study evaluated variability across software tools for IVIM measurements of breast tumors in a heterogeneous multicenter multivendor dataset to test the robustness and diagnostic utility of IVIM biomarkers in a worst-case scenario paradigm. Broadly speaking, *D_t_* metrics present markers of tissue microstructure (especially tumor cellularity) and *f_p_* metrics report on microvascularity. Both of these features are known to be biologically important in determining malignancy and monitoring or predicting response to differently targeted treatment (such as cytotoxic or anti-angiogenic agents). In order to maximize the potential of their separate biologic sensitivities, their numerical robustness must be scrutinized as in the present study.

IVIM parametric maps obtained from different software employing least-squares segmented fitting and Bayesian fitting generated similar *D_t_* and *f_p_* maps. *D_t_* maps were the most consistent across the software platforms at all sites while some differences in *f_p_* maps could be observed particularly at Site B between Software a/b and c. The lower correlations at Site B between *f_p_* values obtained from Bayesian and least-squares packages may have been affected by that site’s low number of b-values sampled in the pseudodiffusion regime (b<200 s/mm^2^); with fewer data constraints Bayesian approaches may regress to their prior. *D_p_* maps were the most variable between the software platforms.

Several *D_t_* radiomic features as well as mean *f_p_* demonstrated high correlations between software pairs. Software correlations were highest between the least squares segmented algorithms (a/b) and mean values are the most consistent across contexts. Multiple *D_t_* radiomic features were highly diagnostic for benign and malignant differentiation as well as consistent across software platforms. However, for *f_p_* metrics, mean and variance, moderately diagnostic on average, were highly consistent among software.

Results of this study indicate some variability in software robustness and benign/malignant differentiation among multi-site data. Some site variability (lesion size, b-value distribution, cohort size, selection criteria) may limit consistency; therefore, a logistic regression model with site adjustment factors was employed to obtain AUCs to account for such heterogeneity in the dataset. LOU AUCs was also derived as a more stringent test of the data, which revealed slight reduction in performance but analogous ranking of parameters. Several *D_t_* metrics showed both software robustness and consistently high diagnostic performance. The robust performance of *D_t_* metrics across different software platforms and sites, particularly for benign/malignant differentiation, supports the potential for widespread implementation of IVIM-DWI beyond its current limited clinical use and research applications (5). On the other hand, several *D_p_* metrics although showing consistency across software platforms were moderately diagnostic on average for benign and malignant differentiation. Several *f_p_* metrics showed only slightly lower diagnostic performance in the logistic regression and were highly consistent across software platforms. These results, obtained in the challenging context of a retrospective analysis of heterogeneous multi-site data, underline the potential additive value of *f_p_* in future prospective multi-site studies.

In general, consistency of *D_t_* radiomic features from least-squares segmented algorithms and Bayesian algorithms agrees with the study conducted by Scalco et al. (35) in that the choice of the quantification method can be neglected for the extraction of 1^st^ order histogram features from *D_t_* maps in case of retrospective multi-center analyses. However, our study also validated that *D_t_* radiomic features obtained from least-squares segmented fitting could be consistent with the Bayesian fitting and therefore the fitting methods for the estimation of *D_t_* maps could be completely neglected. Their study also revealed that *D_p_* is the most sensitive to quantification method and therefore is less robust across software platforms as demonstrated in this study.

Meeus et al. (41) reported that the constrained IVIM fitting method provides robust and reproducible IVIM parameters particularly *D_t_* and *f_p_* in low-perfused brain tissue similar to our study. *D_t_* consistency across software tools reported in the current study was good and in agreement with the reproducibility studies conducted for phantom (42) and kidney (43–46). In addition, we also observed good *f_p_* reproducibility in most contexts.

Our present study had some limitations. Since the study was retrospective there was no control over the differences in acquisition protocols or hardware platforms at different sites; this might be one of many reasons for inconsistency in IVIM parameter maps particularly *D_p_*. There is a possibility that robustness and consistency of *D_p_* maps among software packages was impacted by the different amount of outlier rejection fractions particularly in the case of *D_p_* maps. *D_p_* maps from Firevoxel generated considerably more lesion voxels outside the histogram range (0 – 0.1 mm^2^/s) than did Software b and Software C, which included almost all the lesion voxels. Moreover, the harmonization in b-values would be beneficial for future prospective studies to maintain robustness. Site A in particular may have been affected by heterogeneous sets of b-values and resolution levels within its cohort. The non-Gaussian effect/noise floor was not accounted for in the software used in this study, potentially leading to overestimations of *f_p_* values. While the lesion size among the recruited population in the study cannot be foreseen, however the difference in ROI size in patient population in this retrospective study is also because of the multi-slice segmentation (Site A) or single slice segmentation (Site B and Site C) employed, which could also be the reason for some inconsistency in results. Therefore, uniformity in delineating the lesion must be maintained in addition to recruiting a similar cohort size and consistent recruitment criteria for prospective multicenter studies. Finally, there was some heterogeneity in lesion validation standard (biopsy confirmation at Sites A, B *vs*. radiologic assessment at Site C for benign lesions) in the studied cohorts.

## 5 Conclusion

Even in a heterogeneous multisite cohort with varying acquisition and analysis settings, certain 1^st^ order IVIM radiomic features (specifically mean, minimum and maximum of *D_t_*) show potential for robustness and diagnostic applicability. Pseudodiffusion features (*f_p_* and *D_p_*) are more sensitive to fit algorithms and clinical cohorts, but the mean and variance of *f_p_* still demonstrates potential for consistent behavior among site/software contexts that controlled prospective studies might leverage.

## Data Availability

The datasets presented in this article are not readily available because Data sharing is restricted. The current data use agreement doesn’t allow it to be shared and post publicly. However, upon request, under appropriate institutional data-use agreements’ sharing might be possible. Requests to access the datasets should be directed to DiB, dibash.basukala@nyulangone.org.
