# Supplementary material for: Retrospective BReast Intravoxel Incoherent Motion Multisite (BRIMM) multisoftware study
Source: Front Oncol. 2025 Feb 24;15:1524634. doi: 10.3389/fonc.2025.1524634 (PMC11891049; doi:10.3389/fonc.2025.1524634)
Supplement: Supplementary file 1 [file DataSheet1.pdf]

Supplemental Table S1: Correlation coefficient of IVIM parameters between each Software (a, b, c) pair for 1st order radiomics at each site. Intraclass correlation coefficient (ICC) is given for agreement among three software.

| | 1st order radiomics | $D_t$ | | | | $f_p$ | | | | $D_p$ | | | |
|---|---|---|---|---|---|---|---|---|---|---|---|---|---|
| | | a/b | a/c | b/c | ICC | a/b | a/c | b/c | ICC | a/b | a/c | b/c | ICC |
| Site A | Min | 0.953 | 0.65 | 0.638 | 0.752 | NaN | NaN | NaN | 0 | NaN | 0.006 | NaN | 0 |
| | Max | 0.929 | 0.918 | 0.955 | 0.922 | 0.983 | 0.285 | 0.267 | 0.389 | 0.78 | 0.62 | 0.616 | 0.245 |
| | Mean | 0.998 | 0.984 | 0.989 | 0.99 | 0.992 | 0.826 | 0.841 | 0.873 | 0.914 | 0.573 | 0.501 | 0.456 |
| | Variance | 0.969 | 0.898 | 0.931 | 0.928 | 0.975 | 0.63 | 0.605 | 0.591 | 0.943 | 0.782 | 0.775 | 0.601 |
| | Skew | 0.97 | 0.867 | 0.847 | 0.876 | 0.97 | 0.367 | 0.336 | 0.331 | 0.66 | 0.599 | 0.262 | 0.449 |
| | Kurt | 0.952 | 0.725 | 0.66 | 0.767 | 0.975 | 0.344 | 0.367 | 0.253 | 0.64 | 0.753 | 0.367 | 0.527 |
| | | | | | | | | | | | | | |
| Site B | Min | 0.965 | 0.631 | 0.664 | 0.74 | 0.716 | 0.615 | 0.673 | 0.611 | 0.599 | 0.284 | 0.407 | 0.318 |
| | Max | 0.908 | 0.808 | 0.934 | 0.872 | 0.977 | 0.396 | 0.436 | 0.383 | 0.732 | 0.542 | 0.645 | 0.612 |
| | Mean | 0.986 | 0.949 | 0.971 | 0.95 | 0.931 | 0.704 | 0.789 | 0.642 | 0.82 | 0.581 | 0.541 | 0.582 |
| | Variance | 0.874 | 0.54 | 0.711 | 0.672 | 0.789 | 0.211 | 0.276 | 0.268 | 0.623 | 0.483 | 0.482 | 0.428 |
| | Skew | 0.556 | 0.263 | 0.463 | 0.306 | 0.799 | 0.369 | 0.389 | 0.224 | 0.607 | 0.427 | 0.411 | 0.459 |
| | Kurt | 0.703 | 0.098 | 0.106 | 0.117 | 0.564 | 0.107 | 0.112 | 0.042 | 0.694 | 0.308 | 0.33 | 0.279 |
| | | | | | | | | | | | | | |
| Site C | Min | 0.894 | 0.852 | 0.873 | 0.861 | NaN | -0.023 | NaN | 0 | NaN | 0.191 | NaN | 0.046 |
| | Max | 0.826 | 0.833 | 0.908 | 0.841 | 0.936 | 0.637 | 0.618 | 0.7 | 0.687 | 0.46 | 0.461 | 0.31 |
| | Mean | 0.979 | 0.975 | 0.987 | 0.977 | 0.951 | 0.907 | 0.895 | 0.884 | 0.727 | 0.251 | 0.134 | 0.246 |
| | Variance | 0.814 | 0.722 | 0.876 | 0.796 | 0.951 | 0.861 | 0.817 | 0.858 | 0.592 | 0.289 | 0.315 | 0.215 |
| | Skew | 0.771 | 0.733 | 0.805 | 0.766 | 0.886 | 0.394 | 0.359 | 0.368 | 0.695 | 0.187 | 0.239 | 0.281 |
| | Kurt | 0.782 | 0.622 | 0.605 | 0.663 | 0.918 | 0.262 | 0.248 | 0.158 | 0.763 | 0.369 | 0.366 | 0.346 |

a: Firevoxel; b: Siemens; c: Olea.

$D_t$: tissue diffusivity; $f_p$: Perfusion fraction; $D_p$: pseudo-diffusion

Supplemental Table S2: Bland-Altman analysis of IVIM parameters between each Software (a, b, c) pair for 1st order radiomics at each site.

| | 1st order radiomics | | $D_t$ | | | $f_p$ | | | $D_p$ | | |
|---|---|---|---|---|---|---|---|---|---|---|---|
| | | | a/b | a/c | b/c | a/b | a/c | b/c | a/b | a/c | b/c |
| Site A | Min | Difference Mean | 0.01 | -0.03 | -0.04 | 0 | 0 | 0 | -0.03 | 1.1 | 1.1 |
| | | Difference SD | 0.11 | 0.29 | 0.29 | 0 | 0 | 0 | 0.16 | 0.26 | 0.21 |
| | | CV (%) | 43.73 | 123.8 | 123.62 | NaN | 341.58 | 341.58 | 1095.6 | 44.42 | 36.09 |
| | Max | Difference Mean | -0.1 | -0.08 | 0.03 | -1.69 | 21.68 | 23.37 | -0.85 | -36.4 | -35.6 |
| | | Difference SD | 0.18 | 0.19 | 0.14 | 4.12 | 23.8 | 23.76 | 14.6 | 17.6 | 19.2 |
| | | CV (%) | 7.72 | 8.44 | 6.41 | 7.22 | 34.59 | 34.96 | 18.34 | 28.44 | 31.31 |
| | Mean | Difference Mean | -0.01 | -0.01 | -0.01 | -0.93 | 0.5 | 1.44 | -2 | -4.4 | -2.3 |
| | | Difference SD | 0.03 | 0.07 | 0.05 | 0.99 | 3.58 | 3.19 | 1.4 | 2.9 | 3.1 |
| | | CV (%) | 2.19 | 5.41 | 4.31 | 7.22 | 24.65 | 22.76 | 12.66 | 30.6 | 36.61 |
| | Variance | Difference Mean | 0 | 0 | 0 | -0.03 | 1.21 | 1.24 | 0.01 | -0.06 | -0.07 |
| | | Difference SD | 0 | 0 | 0 | 0.39 | 1.54 | 1.55 | 0.03 | 0.06 | 0.06 |
| | | CV (%) | 17.15 | 29.52 | 24.9 | 23.51 | 68.2 | 69.03 | 20.86 | 54.74 | 55.9 |
| | Skew | Difference Mean | -75.5 | -192 | -116.5 | -9.92 | 85.68 | 95.6 | -536.7 | -569.1 | -32.3 |
| | | Difference SD | 180.2 | 399.3 | 429.6 | 15.76 | 88.89 | 91.11 | 940.2 | 1223.7 | 1463.9 |
| | | CV (%) | 102.85 | 341.31 | 542.2 | 18.68 | 67.26 | 71.63 | 34.94 | 45.75 | 60.84 |
| | Kurt | Difference Mean | -27.4 | 161.4 | 188.8 | -38.36 | 317.78 | 356.14 | -5057.2 | -6458.8 | -1401.6 |
| | | Difference SD | 434.4 | 1115.7 | 122 | 61.99 | 505.97 | 499.14 | 11256.8 | 9980.4 | 13133.5 |
| | | CV (%) | 76.43 | 168.35 | 187.96 | 55.84 | 175.02 | 184.93 | 93.39 | 87.91 | 148.83 |
| | | | | | | | | | | | |
| Site B | Min | Difference Mean | 0.04 | -0.03 | -0.06 | -1.04 | -1.24 | -0.2 | -1.6 | -0.47 | 1.2 |
| | | Difference SD | 0.09 | 0.31 | 0.29 | 3.26 | 3.68 | 2.55 | 1.3 | 1.8 | 1.5 |
| | | CV (%) | 17.63 | 63.46 | 57.51 | 200.26 | 241.07 | 253.54 | 107.66 | 98.13 | 151.29 |
| | Max | Difference Mean | -0.06 | -0.05 | 0.01 | -0.67 | 7.31 | 7.98 | 3.6 | 5.2 | 1.6 |
| | | Difference SD | 0.16 | 0.23 | 0.13 | 2.5 | 23.63 | 23.12 | 8.7 | 11.1 | 9.8 |
| | | CV (%) | 10.67 | 15.22 | 8.51 | 6.05 | 52.24 | 51.48 | 43.15 | 53.18 | 43.08 |
| | Mean | Difference Mean | 0.01 | 0.08 | 0.07 | -1.98 | -7.36 | -5.38 | -0.61 | 0.3 | 0.9 |
| | | Difference SD | 0.06 | 0.1 | 0.08 | 2.78 | 5.7 | 4.86 | 1.8 | 3.1 | 3.4 |
| | | CV (%) | 5.59 | 9.85 | 7.21 | 15.39 | 37.1 | 33.78 | 30.38 | 47.64 | 54.67 |

| | | | | | | | | | | |
|---|---|---|---|---|---|---|---|---|---|---|
| | Variance | Difference Mean | 0 | 0 | 0 | 0.13 | 0.56 | 0.42 | 0.02 | 0.02 | 0 |
| | | Difference SD | 0 | 0 | 0 | 0.55 | 1.82 | 1.76 | 0.03 | 0.03 | 0.04 |
| | | CV (%) | 51.24 | 89.19 | 69.31 | 39.79 | 113.74 | 105.5 | 115.77 | 103.84 | 99.19 |
| | Skew | Difference Mean | -35.9 | -630.9 | -595 | 6.33 | 134.55 | 128.22 | 17.7 | 253 | 235.3 |
| | | Difference SD | 528.9 | 850.6 | 743.9 | 44.03 | 95.87 | 99.43 | 1256.3 | 1519 | 1213.9 |
| | | CV (%) | 2082.9 | -312.59 | -256.45 | 186.35 | 109.26 | 109.38 | 87.09 | 97.36 | 77.36 |
| | Kurt | Difference Mean | -9.3 | 993.5 | 1002.8 | 9.08 | 326.22 | 317.14 | -1949 | -1338.2 | 610.8 |
| | | Difference SD | 664.1 | 2426.4 | 2400.9 | 94.29 | 446.49 | 450.28 | 15438.5 | 17854.7 | 6526.7 |
| | | CV (%) | -202.95 | 1393.1 | 1416.3 | -207.59 | 394.6 | 382.61 | 390.52 | 419.26 | 198.73 |
| | | | | | | | | | | | |
| Site C | Min | Difference Mean | -0.02 | 0.03 | 0.05 | -0.05 | -0.05 | 0 | -0.83 | 0.45 | 1.3 |
| | | Difference SD | 0.16 | 0.19 | 0.16 | 0.45 | 0.45 | 0 | 2.1 | 2 | 0.7 |
| | | CV (%) | 44.54 | 48.52 | 43.62 | 1755.2 | 1755.2 | 999.89 | 494.94 | 193.45 | 108.41 |
| | Max | Difference Mean | -0.08 | -0.13 | -0.05 | -1.74 | 1.29 | 3.03 | -8.5 | -31.8 | -23.3 |
| | | Difference SD | 0.31 | 0.29 | 0.22 | 5.86 | 15.82 | 15.92 | 19.6 | 22.4 | 21.5 |
| | | CV (%) | 17.1 | 16.46 | 12.7 | 14.35 | 37.37 | 38.4 | 31.84 | 44.92 | 47.1 |
| | Mean | Difference Mean | -0.03 | -0.03 | 0 | -1.15 | -1.94 | -0.79 | -6.6 | -6.4 | 0.2 |
| | | Difference SD | 0.07 | 0.08 | 0.06 | 1.73 | 2.33 | 2.35 | 4.6 | 7.2 | 6.1 |
| | | CV (%) | 7.33 | 8.07 | 5.81 | 15.48 | 21.63 | 23.06 | 34.53 | 53.66 | 59.49 |
| | Variance | Difference Mean | 0 | 0 | 0 | -0.08 | -0.06 | 0.02 | -0.14 | -0.26 | -0.12 |
| | | Difference SD | 0 | 0 | 0 | 0.49 | 0.82 | 0.93 | 0.2 | 0.24 | 0.15 |
| | | CV (%) | 56.54 | 73.72 | 50.01 | 32.28 | 53.8 | 62.38 | 71.98 | 107.98 | 99.24 |
| | Skew | Difference Mean | -102.5 | -112.5 | -10 | 1.07 | 46.27 | 45.2 | 311 | -338.3 | -649.4 |
| | | Difference SD | 601.8 | 622.1 | 557.5 | 25.54 | 92.85 | 94.27 | 628.8 | 1297.8 | 1298.2 |
| | | CV (%) | 228.08 | 240.33 | 268.56 | 28.73 | 83.26 | 84.13 | 38.81 | 100.17 | 89.46 |
| | Kurt | Difference Mean | 92.4 | -169.1 | -261.5 | -19.05 | 220.77 | 239.83 | 1687.4 | -283.6 | -1971 |
| | | Difference SD | 1659.1 | 1872.8 | 2134 | 76.88 | 686.88 | 689.41 | 2972 | 7140.6 | 7350.7 |
| | | CV (%) | 184.56 | 243.79 | 262.03 | 211.92 | 439.78 | 470.06 | 105.68 | 390.88 | 275.26 |

a: Firevoxel; b: Siemens; c: Olea.
SD: Standard deviation; CV: Coefficient of variation
Perfusion fraction ($f_p$) is given in %, while pseudo-diffusion ($D_p$) and tissue diffusivity ($D_t$) are given in units of $10^{-3}$ $mm^2/s$.

Supplemental Figure S1: IVIM parametric maps overlaid on raw DWI images in a patient with benign breast lesion for Site A. IVIM parameters tissue diffusivity ($D_t$), perfusion fraction ($f_p$) and pseudodiffusivity ($D_p$) obtained from Firevoxel, Siemens and Olea software in the breast lesion. $D_t$ maps and $f_p$ maps are the most consistent across software platforms, while $D_p$ maps show the most variability with fit algorithms. $D_t$ and $D_p$ are given in units of $10^{-3}$ $mm^2/s$.

Supplemental Figure S2: IVIM parametric maps overlaid on raw DWI images in a patient with benign breast lesion for Site B. IVIM parameters tissue diffusivity ($D_t$), perfusion fraction ($f_p$) and pseudodiffusivity ($D_p$) obtained from Firevoxel, Siemens and Olea software in the breast lesion. $D_t$ maps are the most consistent across software platforms, while $f_p$ and $D_p$ maps show the most variability with fit algorithms. $D_t$ and $D_p$ are given in units of $10^{-3}$ $mm^2/s$.

Supplemental Figure S3: IVIM parametric maps overlaid on raw DWI images in a patient with benign breast lesion (granulomatous mastitis) for Site C. IVIM parameters tissue diffusivity ($D_t$), perfusion fraction ($f_p$) and pseudodiffusivity ($D_p$) obtained from Firevoxel, Olea and Siemens software in the breast lesion. $D_t$ maps and $f_p$ maps are the most consistent across software platforms, while $D_p$ maps show the most variability with fit algorithms. $D_t$ and $D_p$ are given in units of $10^{-3}$ $mm^2/s$.

Supplemental Figure S4: Bland-Altman plots between Firevoxel, Siemens and Olea for mean of tissue diffusivity ($D_t$) at Site A, Site B and Site C. Comparisons shown left to right: Firevoxel and Siemens, Firevoxel and Olea, Siemens and Olea. RPC is reproducibility coefficient.

Supplemental Figure S5: Bland-Altman plots between Firevoxel, Siemens and Olea for mean of perfusion fraction ($f_p$) at Site A, Site B and Site C. Comparisons shown left to right: Firevoxel and Siemens, Firevoxel and Olea, Siemens and Olea. RPC is reproducibility coefficient.
