# Supplementary figures and images for: Retrospective BReast Intravoxel Incoherent Motion Multisite (BRIMM) multisoftware study
Source: Front Oncol. 2025 Feb 24;15:1524634. doi: 10.3389/fonc.2025.1524634 (PMC11891049; doi:10.3389/fonc.2025.1524634)

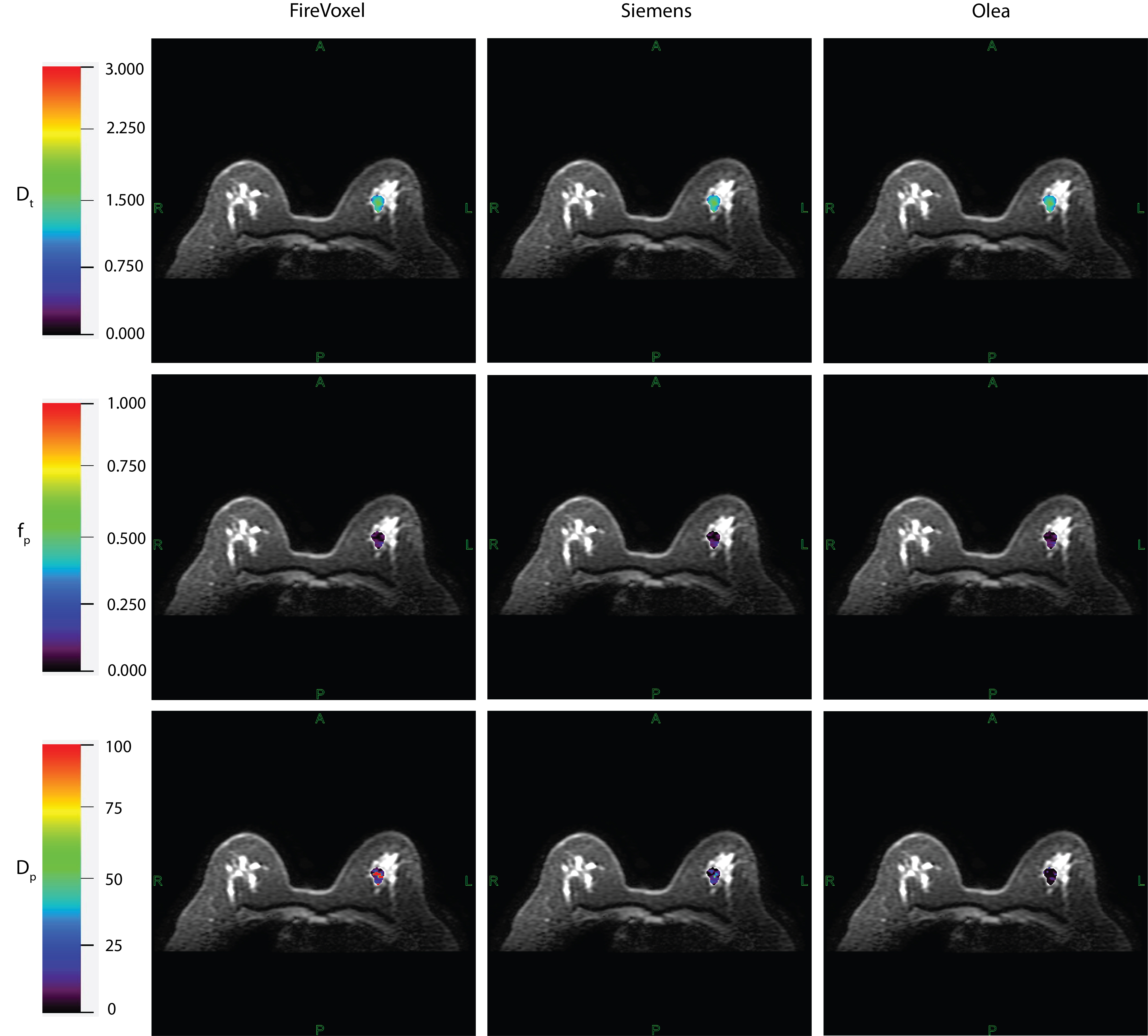

Supplement: Supplementary file 2 [file Image1.jpeg]

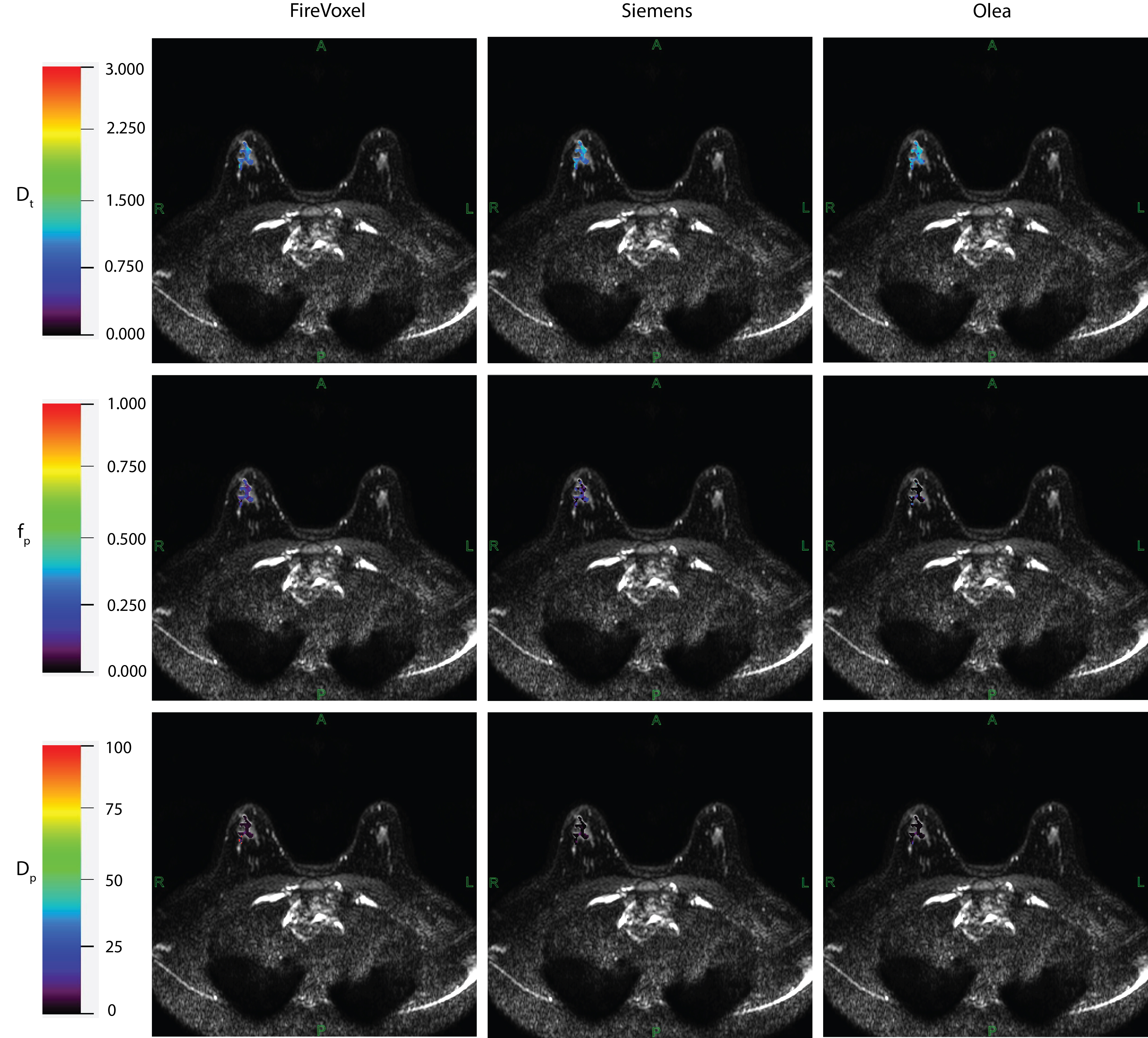

Supplement: Supplementary file 3 [file Image2.jpeg]

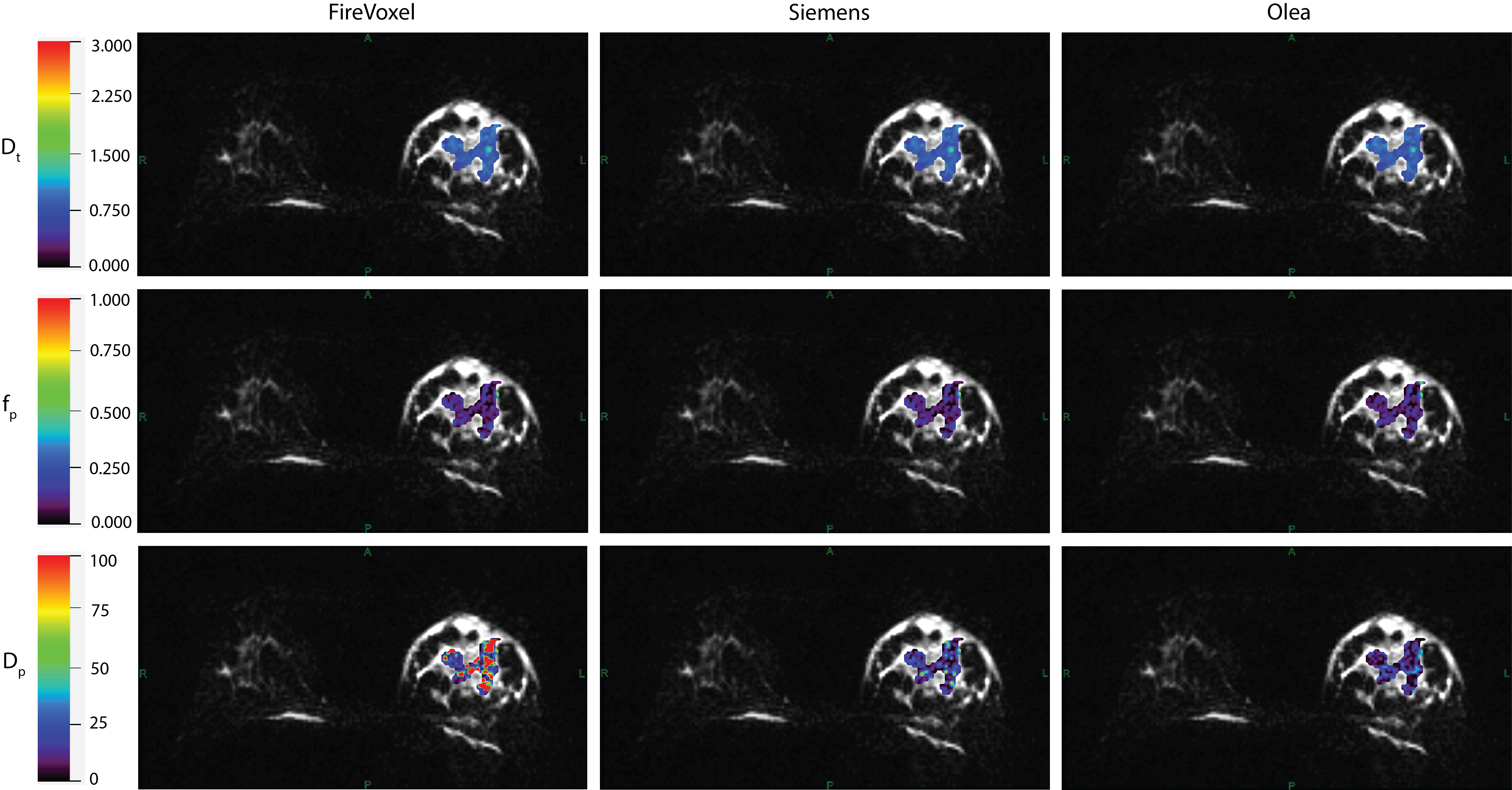

Supplement: Supplementary file 4 [file Image3.jpeg]

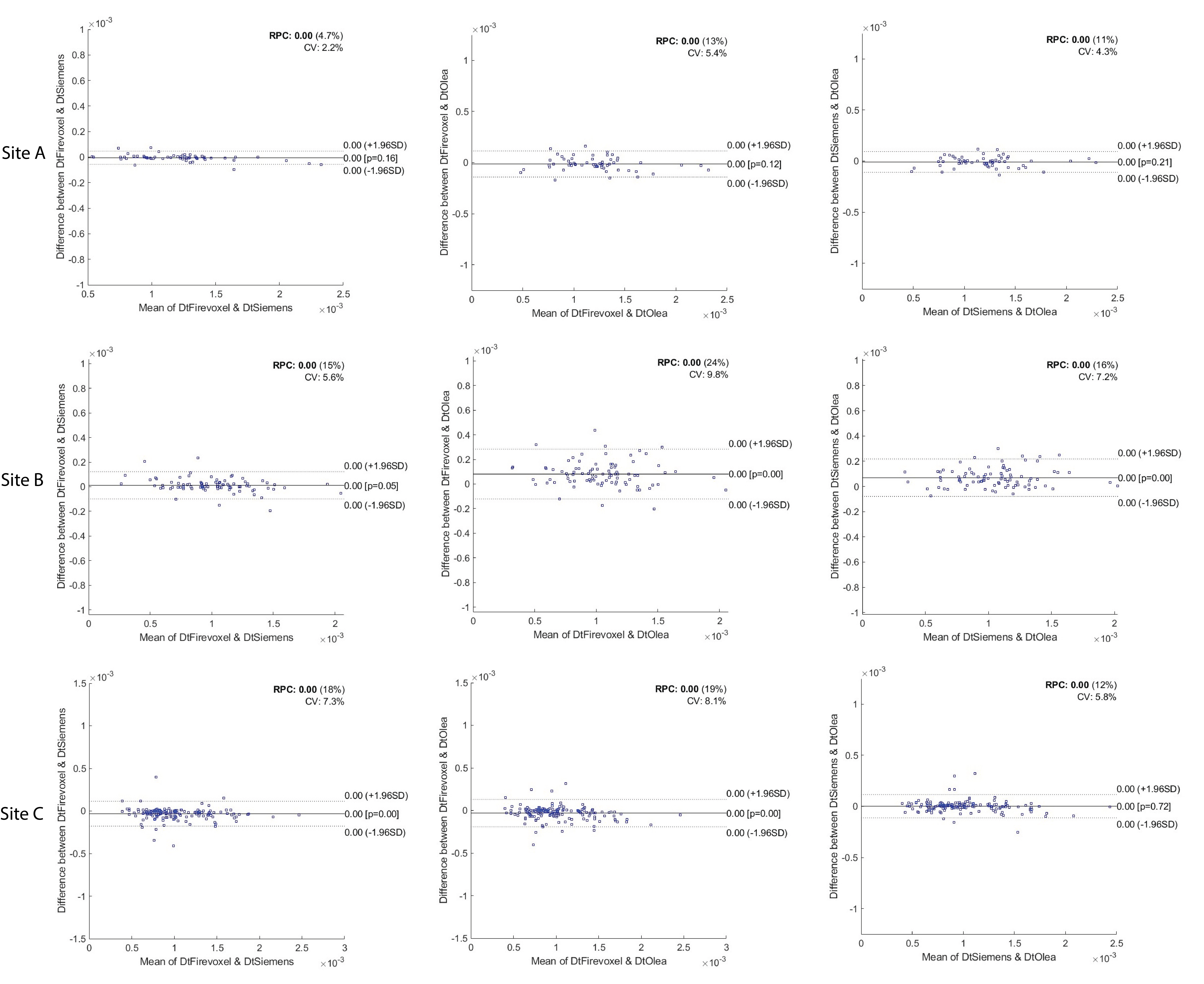

Supplement: Supplementary file 5 [file Image4.jpeg]

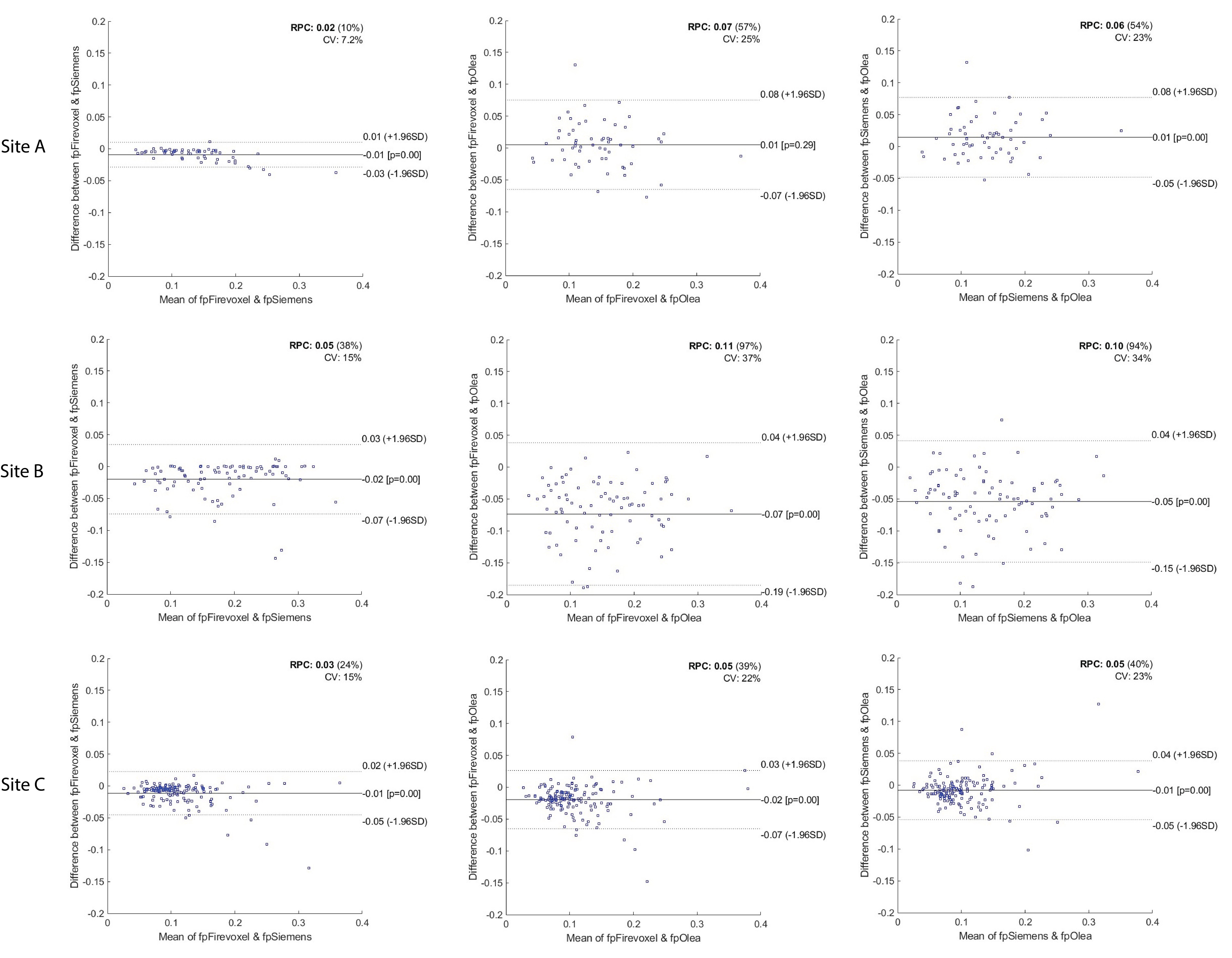

Supplement: Supplementary file 6 [file Image5.jpeg]
